# Cutaneous Protothecosis in a Patient with Chronic Lymphocytic Leukemia: A Case Report and Literature Review 

**DOI:** 10.3390/jof1010004

**Published:** 2015-01-14

**Authors:** Quynh-Giao Ly Nguyen, Ted Rosen

**Affiliations:** Department of Dermatology, Baylor College of Medicine, 1977 Butler Blvd, Suite E6.200, Houston, TX 77030, USA; E-Mail: quynhgin@bcm.edu

**Keywords:** protothecosis, *Prototheca wickerhamii*, achlorophyllic algae

## Abstract

Protothecosis is a rare infection, which has the potential to cause severe disease in patients with underlying immunosuppression. We describe a case of an elderly female with chronic lymphocytic leukemia (CLL), as well as other risk factors, who presented with pustular and erythematous plaques, initially presumed to be leukemia cutis. A biopsy with special stains revealed the lesions to be cutaneous protothecosis, thus presenting a most unusual concurrence of disease entities. The literature to date on this rare infection will be reviewed.

## 1. Introduction

Protothecosis is a rarely-reported infection caused by achlorophyllous algae of the *Prototheca* genus, derived from the common green algae, *Chlorella* [1,2]. In addition to lacking chloroplasts, and, thus, chlorophyll, *Prototheca* also differ from *Chlorella* in that they have neither galactose nor galactosamine in the cell wall. A final differentiating feature between *Prototheca* species and *Chlorella* is that the former possess a two-layered, instead of a three-layered, cell wall as seen by electron microscopy [2]. The precise taxonomy of the genus remains somewhat debatable and has been the subject of multiple prior reclassifications. At least three distinct species appear to be universally accepted: *P. wickerhamii*, * P. zopfii* and *P. stagnora*. *Prototheca* are found widely in nature, though they thrive in environments, such as stagnant water, slime flux and animal waste. The cutaneous infection is most common in patients with underlying immunosuppression or in association with several underlying systemic and debilitating diseases following traumatic inoculation [3,4,5]. Herein, we describe a case of cutaneous protothecosis in an elderly female with multiple comorbidities, including chronic lymphocytic leukemia, and review the pertinent and readily-available literature on this subject. 

## 2. Case Report

**Figure 1:**
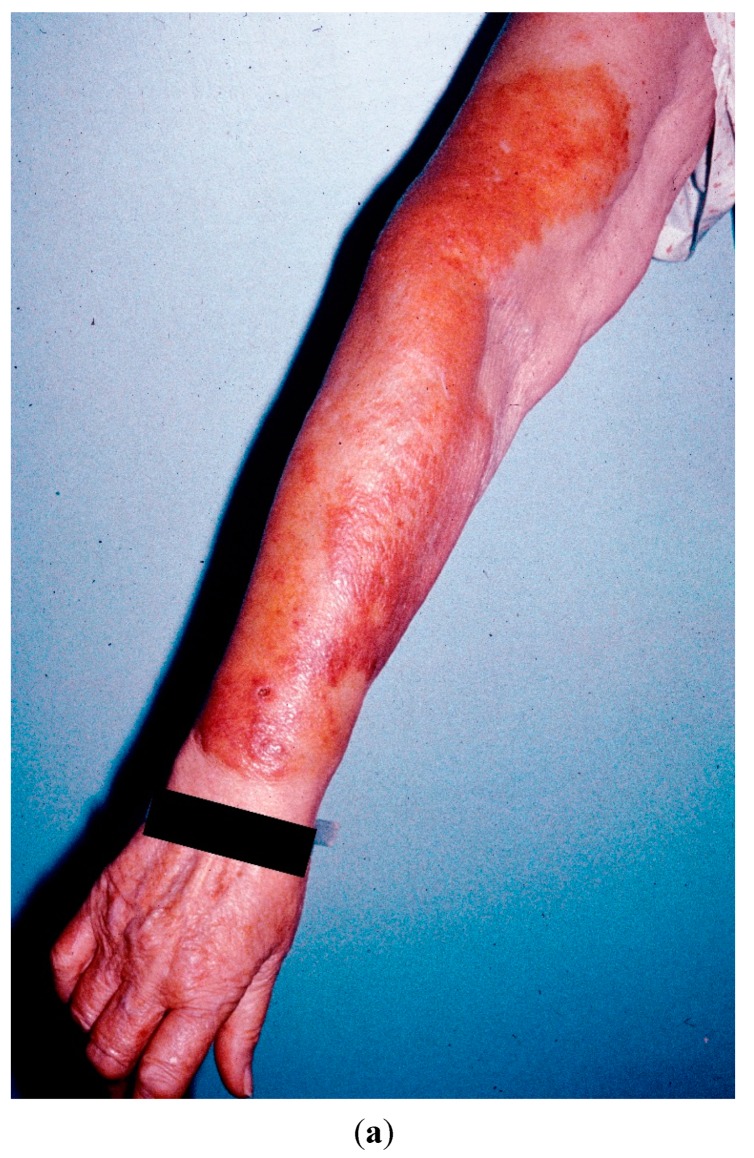

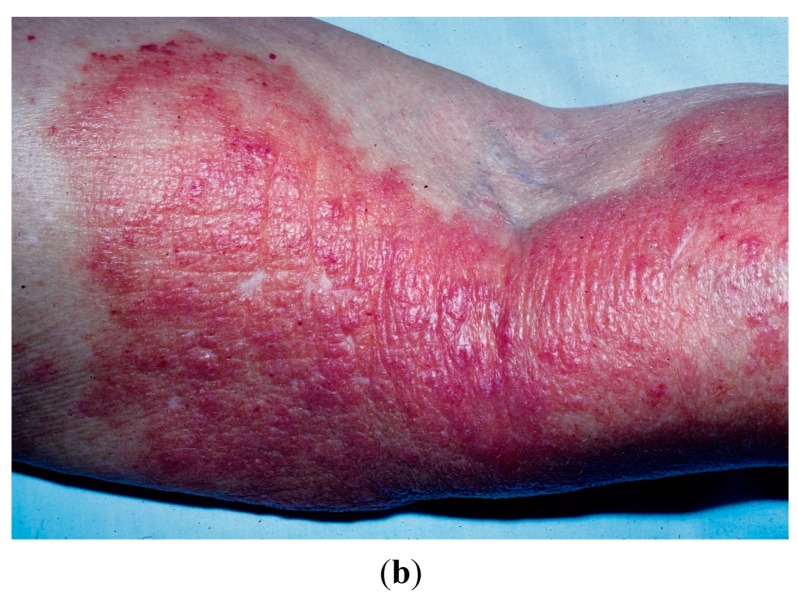
(**a**) Indurated erythematous plaques on the dorsal forearm; (**b**) close view of the indurated plaque.

A 72-year-old female with a past history of bilateral mastectomy due to intraductal breast cancer ten years previously, insulin-dependent and poorly-regulated diabetes mellitus, moderate hypertension, partially-compensated congestive heart failure, and, most recently, chronic lymphocytic leukemia (CLL) underwent dermatologic evaluation due to the presence of asymptomatic, small, pustular and larger erythematous plaques that progressively developed over a period of three months. The skin lesions preferentially involved the dorsal surface of both upper extremities. (Figure 1a,b).

**Figure 2:**
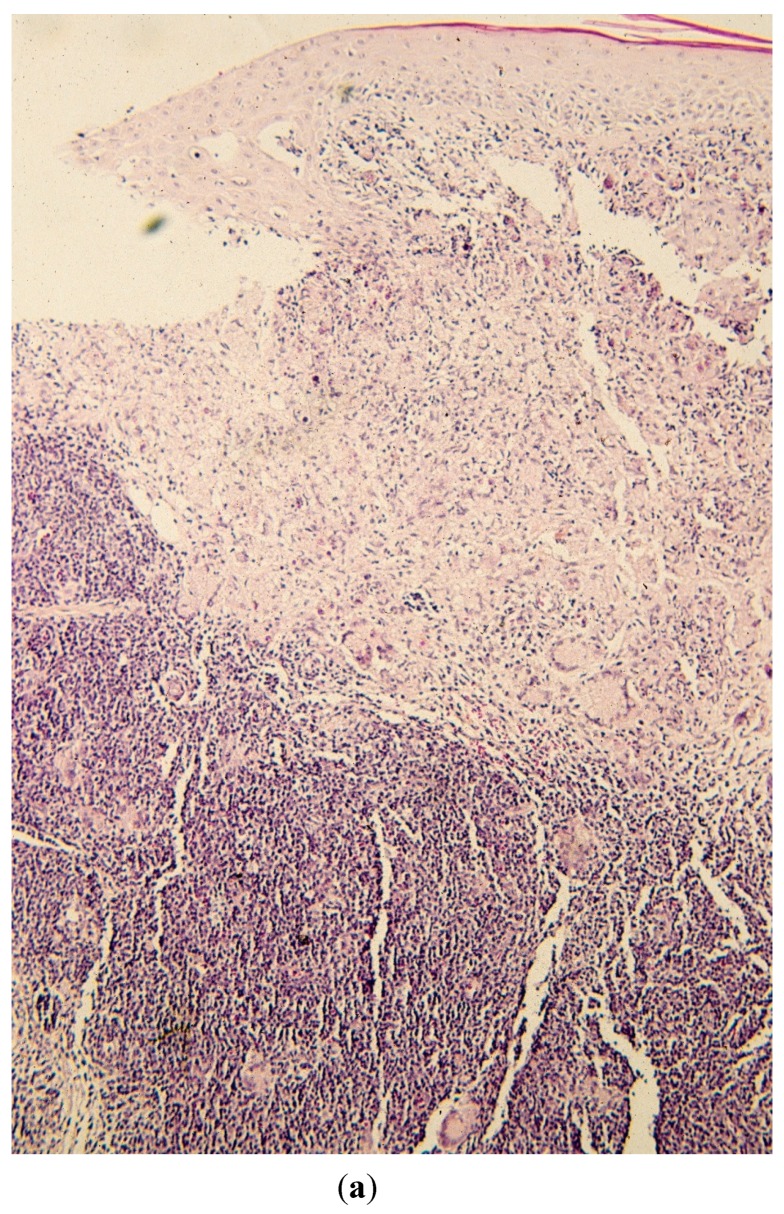

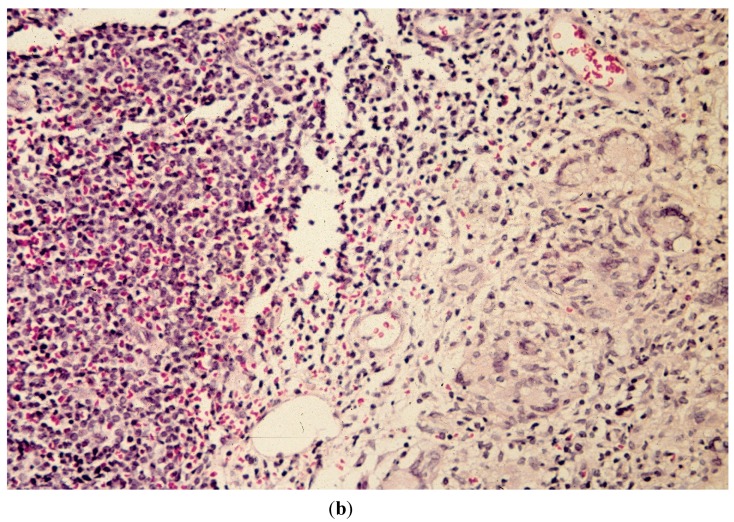
(**a**) Biopsy demonstrating both leukemic infiltrate and granuloma formation within the dermis, the latter being located more superficially (hematoxylin-eosin stain at 50×); (**b**) closer view of the biopsy demonstrating both leukemic infiltrate and granuloma formation with prominent multinucleated giant cells (Hematoxylin-eosin stain at 125×).

She had been diagnosed with CLL approximately five years earlier, and her treatment regimen included cyclical administration of cyclophosphamide, vincristine and prednisone. The latter was given in daily oral doses of 40 mg. The patient was generally well-appearing, but hemodynamically unstable, with white cell counts ranging between 20,000 and 180,000/mL^3^. Biopsies of several plaques were performed and special stains requested to rule out various infectious processes.

Histopathologic examination in hematoxylin and eosin (H&E)-stained sections revealed concurrent and adjacent leukemic infiltrate consisting of mature lymphocytes and granulomata with obvious giant cells (Figure 2a,b).

**Figure 3:**
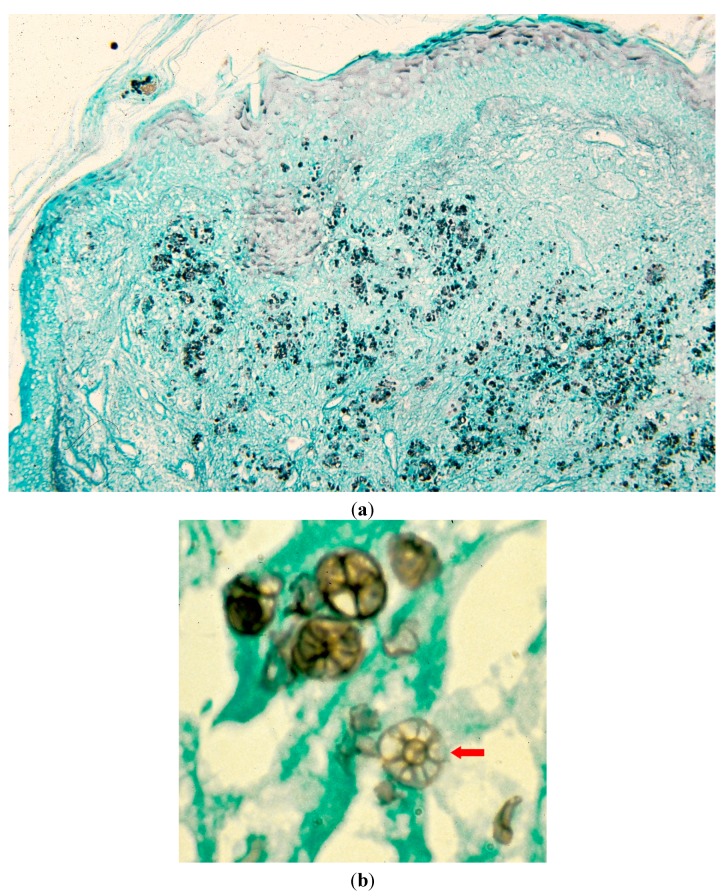
(**a**) Numerous organisms stained with Gomori methenamine silver (GMS) in tissue (GMS stain at 50×); (**b**) the arrow points to the typical “floret” appearance of *P. wickerhamii* (GMS stain at 250×).

Bodies of microorganisms visible on H&E were then highlighted by Gomori methenamine silver (GMS), periodic acid-Schiff (PAS) and Alcian blue stains. These organisms consisted of a single round cell with multiple radial septations (Figure 3a,b). Upon culturing, both a tissue specimen and a pustule, mucoid beige colonies developed on Sabouraud’s dextrose agar, with sporangia visualized within the cells. A diagnosis of protothecosis was established. The microorganism was ultimately identified as *Prototheca wickerhamii* on the basis of morphological characteristics and sugar assimilation tests. There was no evidence of systemic dissemination.

The patient received a combination of oral tetracycline and intravenous amphotericin-B as the treatment. However, she succumbed to complications from her other co-morbidities before the treatment could reasonably have been expected to succeed. 

## 3. Discussion 

Protothecosis represents an extremely rare opportunistic infection, most commonly encountered in dogs, cats and cattle in whom it may cause mastitis, diarrheal enteritis, blindness and seizures. It is caused by organisms in the *Prototheca* species, most commonly in humans, due to the species *P. wickerhamii* (as in our patient). The other specific species reported to cause human disease is *P. zopfii*. The infection typically manifests in one of three forms: intra-cutaneous to subcutaneous nodules and plaques, olecranon bursitis and systemic (disseminated) disease [6]. Our patient exhibited lesions on the extensor surface of the upper extremities, representing dermal disease. Skin lesions of protothecosis manifest in a heterogeneous and, therefore, non-specific manner. Reports of papules, pustules, plaques, nodules, ulcers, crusts, erosions or even eczematous, herpetiform, granulomatous and verrucous lesions all have been documented in the medical literature [7,8,9]. The lesions of cutaneous protothecosis generally present on the extremities and/or the face [10,11,12,13], though on rare occasions have appeared on the anterior or posterior torso [14,15].

The nature of immune defects associated with and presumably facilitating this unusual infection are unclearly defined. It is suspected that neutrophilic dysfunction and impaired humoral immunity play a major role in protothecosis development [16,17,18]. It is also theorized that sufficient suppression of T-cell-mediated immunity may also play a role in disease acquisition or dissemination [15,19]. Most protothecosis infections occur in patients with predisposing factors, such as human immunodeficiency/acquired immunodeficiency syndrome, diabetes mellitus, underlying malignancy, chemotherapy, radiotherapy, renal transplantation and prolonged systemic corticosteroid administration [20,21,22]. Of approximately ninety-four total reports of cutaneous protothecosis, the most common risk factors appear to be diabetes mellitus (21%) and chronic steroid use (24%) [6,9,23,24,25,26,27]. Malignancy is associated in only 9% of cases; however, the presence of multiple other risk factors in conjunction with neoplasia warrants the consideration of protothecosis in the differential diagnosis when puzzling non-specific skin lesions are detected. Our patient had not only active CLL (and associated multi-agent chemotherapy, including systemic steroids), but also a history of poorly-controlled diabetes, previous breast cancer, hypertension and congestive heart failure. We posit that her multiple chronic diseases may have played a role in leading to protothecosis. Interestingly, neither a history of exposure to stagnant water or animal waste, nor a history of traumatic lesions could be obtained.

Because clinical findings can be so nonspecific from a morphological standpoint, the diagnosis of protothecosis is typically made by histopathologic examination, followed by microbiologic confirmation. Cutaneous biopsies characteristically reveal pan-dermal, granulomatous inflammatory infiltrate, often admixed with neutrophils, eosinophils, plasma cells and/or giant cells. Necrosis within the granulomas and/or pseudocarcinomatous epidermal hyperplasia may be observed [22,25]. Diagnostic spherical sporangia containing symmetrically-distributed endospores confer a berry-like or flower-like (“floret”) appearance, best appreciated with special stains, including periodic acid-Schiff (PAS) and Gomori methenamine-silver (GMS) stains [22,28]. This “morula” appearance is characteristic of *P. wickerhamii*, but not of other *Prototheca* [2]. The culture of the organism in routine mycological media between 25 °C and 37 °C can provide a definitive diagnosis based upon differential rates of sugar assimilation (fructose, glucose, sucrose, galactose), metabolic response to blue light irradiation and analysis of rRNA sequences [2,29]. Despite these diagnostic maneuvers, protothecosis can still be difficult to diagnose due to some similarity to the following fungi: *Blastomyces dermatitidis*, *Coccidioides immitis*, *Cryptococcus neoformans*, *Paracoccidioides brasiliensis* and *Rhinosporidium seeberi* [22,30]. Thus, when the clinical suspicion for protothecosis is high, it is worthwhile to enlist the aid of a mycologist.

Although a treatment regimen has not been standardized, successful options for cutaneous lesions have included amphotericin B, ketoconazole, itraconazole, fluconazole and tetracyclines, all with or without surgery [10,13,15,18,31,32]. There is a single report of the successful use of caspofungin in the literature [33]. Successful use of local thermotherapy (heat) as an adjunct to itraconazole therapy has also been reported [34]. More recently, a newer antifungal agent, voriconazole, has become another potential option for the treatment of cutaneous protothecosis, as it has fewer potential hepatotoxic side effects than several of the other azole agents [23]. There is no widely-accepted duration of therapy, the latter being continued until clinical resolution is achieved.

## 4. Conclusions 

In summary, we report an unusual case of cutaneous protothecosis in a patient with CLL and multiple other risk factors. It is of note that very few cases have been documented in the medical literature of CLL and protothecosis infection. This may suggest that *Prototheca* spp. have low pathogenic potential in these particular cancer patients. Regardless, protothecosis should be included in the presumptive differential diagnosis of cutaneous infection in any cancer patient with other predisposing factors, particularly diabetes and chronic steroid use.
